# Formyl-methionyl-leucyl-phenylalanine Induces Apoptosis in Murine Neurons: Evidence for NO-Dependent Caspase-9 Activation

**DOI:** 10.3390/biology8010004

**Published:** 2019-01-04

**Authors:** Chiara Porro, Antonia Cianciulli, Teresa Trotta, Dario Domenico Lofrumento, Rosa Calvello, Maria Antonietta Panaro

**Affiliations:** 1 Department of Clinical and Experimental Medicine, University of Foggia, 71100 Foggia, Italy; chiara.porro@unifg.it (C.P.); teresa.trotta@unifg.it (T.T.); 2 Department of Biosciences, Biotechnologies and Biopharmaceutics, University of Bari, Via Orabona, 4, 70126 Bari, Italy; antonia.cianciulli@uniba.it (A.C.); rosa.calvello@uniba.it (R.C.); 3 Department of Biological and Environmental Sciences and Technologies, Section of Human Anatomy, University of Salento, 73100 Lecce, Italy; dario.lofrumento@unisalento.it

**Keywords:** formyl-methionyl-leucyl-phenylalanine, apoptosis, neurodegeneration, nitric oxide, neurons, caspase

## Abstract

Formyl-methionyl-leucyl-phenylalanine (fMLP) may be present in the brain in the course of some infectious diseases of the central nervous system (CNS), although little is known about its role. This investigation was performed to study the effect of fMLP on neuron apoptosis. Our results showed that fMLP treatment of primary cultures of neurons was able to induce morphological features of apoptosis in cell cultures, as well as activation of the intrinsic apoptotic pathway, through the upregulation of caspase-9 and caspase-3. This effect contextually occurred to the pro-apoptotic protein Bax activation and cytochrome c release. The in vitro fMLP treatment was also able to induce, in a dose-dependent manner, the increase of inducible nitric oxide synthase (iNOS) expression accompanied by an up-regulation of nitric oxide (NO) release. When neuron cultures were pre-treated with 1400 W, a selective iNOS inhibitor, all of the apoptotic features were significantly reversed. Overall, these results demonstrated that fMLP treatment of neurons leads to intrinsic apoptosis activation, through iNOS expression regulation, suggesting a role for fMLP in CNS neurodegenerative processes.

## 1. Introduction

N-formylated peptides such as *N*-formyl-l-methionyl-l-leucyl-phenylalanine (fMLP) act as potent chemoattractants. These molecules derive from either degraded bacterial or mitochondrial proteins, and play a key role in defense mechanisms toward microbial infection or tissue injury by phagocyte engagement to the inflammation site [1]. Peptides containing a formylated methionine in their N-terminus may represent a ‘‘molecular pattern” identified by cells. These molecules are often linked not only with bacterial infections, but also with danger signals deriving from damaged host cells/tissues, thus highlighting the importance for formyl peptide receptors (FPRs) in inflammatory responses [2,3]. fMLP is revealed to be present in some brain infectious diseases, as meningitis. fMLP released from bacteria could gain access to the brain from the bloodstream through less tight blood–brain barrier (BBB) areas, represented, for example, by the circumventricular organs [4]. Since during inflammatory events BBB function may be compromised, neuronal dysfunction can worsen [5].

Molecular mechanisms that may induce BBB dysfunction include NADPH oxidase or nitric oxide (NO) synthase activation as well as macrophage/microglial activation. These events can lead to the generation of reactive species [6]. Many lines of evidence suggest the presence of FPRs in the human brain, in particular, in neurons [7]. Moreover, it was reported that FPR2 is localised in the rat central nervous system [8]. 

NO, a non-typical neurotransmitter, participates in the physiological activities of neural cells contributing to maintain homeostasis of normal brain. It is a regulator of neurogenesis and synaptogenesis, allowing electrical transmission between neurons and ensuring the efficiency of synaptic contacts [9]. 

About neurotoxic effects, a number of studies have described NO as a critical regulator of neuroinflammation during neurodegenerative and neurotoxicity diseases, thus suggesting its implication in the major brain disorders. Then, positive or negative effects of NO are dependent on different signal pathways or cellular origins and locations. In the present study, we investigated the possible role for the fMLP in neurodegeneration, using a well-established primary culture of neuron represented by rat E18 primary hippocampal neurons [10]. In particular, the aim of this study was to focus attention on the possible effects of formylated bacterial products, such as fMLP, on the neuronal response, evaluating possible neurotoxic effects in terms of cell apoptosis using an experimental model represented by rat hippocampus cell cultures stimulated with fMLP.

Results of this study highlight for the first time the ability of fMLP to induce apoptosis through NO-mediated caspase-9 activation in primary neurons, suggesting that the presence of bacteria derived molecules in the brain may contribute to the neuronal death observed in neurodegenerative diseases.

## 2. Material and Methods

### 2.1. Primary Neuron Cultures

For this study we used cultures of rat hippocampus cells isolated from embryos at 18 days, (Cat# PC 35101) purchased from Neuromics Inc. (Società Italiana Chimici, Rome, Italy). Cells were plated at a density of 25 × 10^5^/15-mm well (ThermoFisher Scientific, Milan Italy) on glass coverslips in presence of 10 μg/mL poly-D-lysine were cultured to get neuron-enriched cultures. Neurobasal medium supplemented with B27 (GIBCO, ThermoFisher Scientific, Milan Italy ), 0.5 mM glutamine, 25 μM glutamate, Penicillin/Streptomycin (100 units/100 μg for mL) and an antimycotic agent, Amphotericin B was used for the maintenance of cell cultures. 

Half-volume medium was changed the day after cell plating, whereas the medium was routinely completely replaced every 3 days. Neuron culture was maintained at 37 °C and 5% CO_2_. After 8 days cells were submitted to the treatments.

### 2.2. Morphologic Analysis

We determined, by immunofluorescence, the FPR2 expression on cell membrane of neuron cultures. Cells were fixed with 4% paraformaldehyde (PFA) for 15 min and then blocked with goat serum at 5% for 1 h at room temperature (RT). Then, cells were incubated for 1 h at RT with rabbit polyclonal anti-FPR2 antibody (Ab) (1:200 diluted in BSA 1%) (ThermoFisher Scientific, Milan, Italy). At the end, 1 h incubation at RT with the secondary goat FITC-conjugated Ab (1:1000 diluted in BSA 1%) (Molecular Probes, Invitrogen, Milan, Italy) was performed. For neuron identification, cells were simultaneously incubated with monoclonal (MoAb) anti-neurofilament 68 mouse IgG (1:200 diluted in BSA 1%) (Sigma-Aldrich, Milan, Italy), specific for cytoskeletal proteins of neurons, and subsequently incubated with the secondary goat TRITC-conjugated Ab (1:1000 diluted in BSA 1%) (Molecular Probes, ThermoFisher Scientific, Milan Italy ). Nuclei were stained with 4,6-diamidino-2-phenylindole. For cell observation a confocal microscope Leica 63 by oil immersion lens was used.

### 2.3. NO Production

A Griess reaction was used to evaluate nitrite concentration as a stable end-product of NO metabolism. Supernatants obtained from cells, treated with different concentrations of fMLP (10^−7^, 10^−9^, 10^−11^, 10^−13^, 10^−15^ M) (Sigma-Aldrich, Milan, Italy) for 48 h, were mixed with the Griess reagent (1:1 *v*/*v*) for 10 min at room temperature and submitted to a spectrophotometric measurement at 550 nm. A sodium nitrite standard curve expressed as nmol/mL was utilised to determine nitrite concentrations in the culture media.

### 2.4. Annexin V-CY3 Assay

Apoptotic cells percentage evaluation has been carried out using the Annexin V-CY3 (AnnCy3) detection kit (Sigma-Aldrich), by simultaneously incubation with both AnnCy3 and 6-CFDA according to manufacturer’s instructions. After labelling, cells were observed by fluorescence microscopy using a 530 nn filter for AnnCy3, and a 455 nm filter for 6-CFDA, respectively. The apoptosis (green cells) percentage was determined by counting at least a total of 300 cells and results were expressed as mean ± SD of five different experiments.

### 2.5. DNA Fragmentation Assay

As an index of apoptosis we also assessed DNA fragmentation analysis. For this purpose, 10^7^ cells from each treatment were firstly washed in PBS and then lysed in 10 mM Tris pH 7.4, 5 mM EDTA, 1% (*v*/*v*) Triton X-100 (all from Sigma-Aldrich) for 20 min on ice. For DNA extraction 1 mL of Trizol ThermoFisher Scientific, Milan Italy) was added to cells according to the manufacturer’s instructions. The extracted DNA was then precipitated in 100% ethanol, centrifuged at 11,000 *g* for 20 min, separated in a 1.8% (*w*/*v*) agarose gel and then visualized by gel-red (Biotium, Società Italiana Chimici, Rome, Italy) staining.

### 2.6. Caspase-3 Enzymatic Activity

The quantitative measurement of caspase-3 (DEVDase, ThermoFisher Scientific, Milan Italy) protease activity in lysates of neuronal cells was assessed using the CaspACE™ colorimetric assay system (Promega, Milan, Italy), that provides the colorimetric substrate DEVD (Ac-Asp-Glu-Val-Asp-7-amino-4-trifluoromethylcoumarin), labelled with the chromophore p-nitroanilide (Ac-DEVD-pNA). 

Briefly cells treated as above described were washed twice in pH 7.2 ice-cold PBS and lysed according to the manufacturer’s instructions. The protein lysates obtained were incubated at 37 °C for 4 h in the presence of DEVD-pNA in flat-bottomed microtiter plates and then caspase-3 activity was detected measuring the p-nitroanilide (pNA) absorbance at 405 nm. The specificity of the reaction was assessed adding to sample the competitive inhibitor of caspase-3, Z-valyl-alanyl-aspartic acid–fluoromethyl ketone (Z-VAD-FMK) (20 mM).

### 2.7. Caspase-8 and Caspase-9 Enzymatic Activity

On neuronal cells lysates, the enzymatic activity of caspase-8 and caspase-9 was also evaluated using colorimetric protease assay kits (BioSource International, ThermoFisher Scientific, Milan Italy ) in which is included the synthetic tetrapeptide Ile–Glu–Thr–Asp (IETD) conjugated to pNA that acts as substrate for active caspase-8, as well as the Leu–Glu–His–Asp (LEHD) pNA-conjugated amino acid sequence which is a substrate for active caspase-9. In brief, after neuronal lysate preparation, 100 μg of proteins were transferred tojl a 96-well microtiter plate, mixed with IETD-pNA or LEHD-pNA and incubated at 37 °C for 2 h. Free pNA light absorbance was measured at 405 nm. Enzymatic activity was also evaluated after cell pre-treatment (1 h) with the caspase-8 inhibitor Z-IETD-FMK or the caspase-9 inhibitor Z-LEHD-FMK.

### 2.8. Electrophoresis and Western Blotting Analysis

Lysis buffer (1% Triton X-100, 20 mM Tris–HCl, 137 mM NaCl, 10% glycerol, 2 mM EDTA, 20 μM leupeptin hemisulfate salt, mM phenylmethylsulfonyl fluoride (PMSF), 0.2 U/mL aprotinin (Sigma–Aldrich)) was used to obtain cell lysate. Briefly, neurons were mixed with lysis buffer for 30 min on ice, vortexed for 15–20 s and then centrifuged at 12,800 *g* for 20 min. For cytosolic cytochrome (Cyt)-c evaluation cells submitted to different treatments were lysed in the presence of the lysis buffers provided by a specific Mitochondria/Cytosol Fractionation Kit according to manufacturer’s instructions (Abcam, Cambridge, UK). A Bradford protein assay was performed to determine total protein content. Protein samples, diluted with sample buffer (0.5 M Tris HCl pH 6.8, 5% b2-mercaptoethanol, 10% *w*/*v* SDS, 10% glycerol, 0.05%, *w*/*v* bromophenol blue) and boiled for 3 min, were then loaded (25 μg/lane) on 7% SDS precast polyacrylamide gels (BioRad Laboratories, Hercules, CA, USA) together with molecular weight prestained standards (BioRad Laboratories), submitted to electrophoresis and then transferred from the gel to nitrocellulose membranes using a blotting buffer [20 mM Tris/150 mM glycine (pH 8), 20% (*v*/*v*) methanol]. Membranes were gently shaken for 1 h with a blocking solution (bovine serum albumin (BSA), 0.2–5% (*w*/*v*), Tween-20 (0.05–0.1%), Casein (1%), non fat dry milk (0.5–5%) (BioRad Laboratories)) to avoid nonspecific binding. The membranes were then incubated in the dark with primary antibody (1:200 diluted): iNOS, caspase-3, -8, -9, Bcl-2, Bax, and Cyt-c (all from Santa Cruz Biotechnology, Heidelberg, Germany), FPR2 (ThermoFisher Scientific, Milan, Italy) overnight at 4 °C. The day after, membranes were washed twice with T-PBS (for 20 min, 3 times) and, immediately after, incubated with the secondary antibody (1:2000) horseradish peroxidase (HRP)-conjugate (Santa Cruz Biotechnology) for 60 min. Bands were visualized using chemiluminescence (BioRad, Laboratories). The 1D Image Analysis Software (Kodak Digital Science, Sigma-Aldrich, Milan, Italy) was used to perform semi-quantitative analysis of visualized bands and results were reported as arbitrary units.

### 2.9. Data Analysis

Parametric (ANOVA/Tukey) and nonparametric (Kruskal–Wallis/Dunn’s post hoc) tests were performed, in accordance with the data distribution to compare the results. A *p* value of < 0.05 was considered statistically significant, and indicated with (*).

## 3. Results

### 3.1. Expression of FPR2 Protein in the Rat Primary Neurons

**Figure 1:**
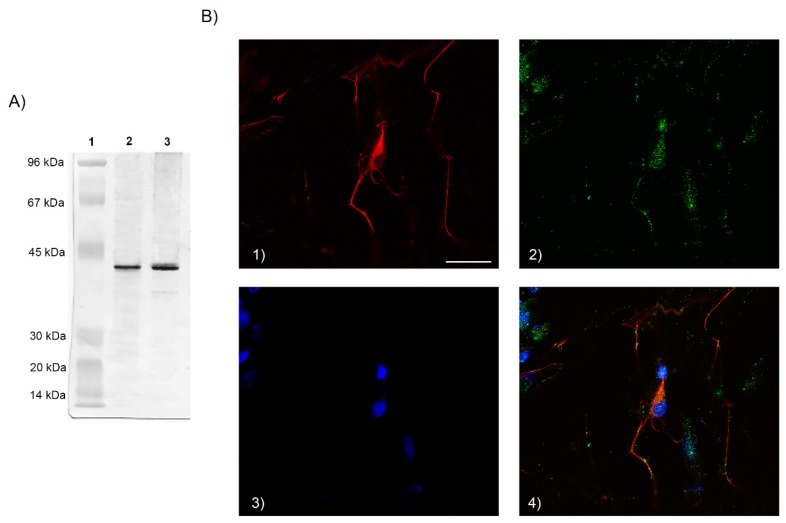
(**A**) Western blot analysis was performed on membrane enriched cell extracts (25 µg lysate) of primary neurons. The blots were probed with formyl peptide receptor 2 (FPR2) antibody (Ab) and detected by chemiluminescence. A ~41 kDa band corresponding to FPR2 was observed as compared to the positive control. Lane 1: Marker; lane 2: Positive control, lane 3: Primary neuron lysate. (**B**) Immunofluorescence identification of FPR. Double staining shows the expression of the FPR receptor on cell membrane and neurofilaments. FPR2 expression (green); skeleton protein staining of neuron-specific neurofilament 68 (red); DAPI nuclear staining (blue); cells stained by both neurofilament 68, FPR2 and DAPI (merged). Scale bar: 100 μm. 1): neurofilaments stain; 2) FPR2 expression; 3) DAPI stain; 4) merged.

Western blot analysis showed that the FPR2 antibody recognised a specific band as reported in Figure 1A, indicating specificity of the antibody; the 41 kDa band corresponds to the molecular weight of FPR2. Rat brain normal tissue (Abcam, Milan, Italy) was used as a positive control.

When cells were examined at confocal microscope after incubation with Ab specific for FPR2, followed by treatment with a goat antimouse IgG TRITC-conjugated secondary Ab, a positive labelling at cell membrane level was detected (Figure 1B), whereas no signal was observed at intracellular level. 

Taken together, these results confirm the expression of the FPR2 on cell membrane of rat neuron primary cultures used in this experimental model.

### 3.2. Expression of Inducible Nitric Oxide Synthase (iNOS) and NO Release in the fMLP Stimulated Cells

**Figure 2:**
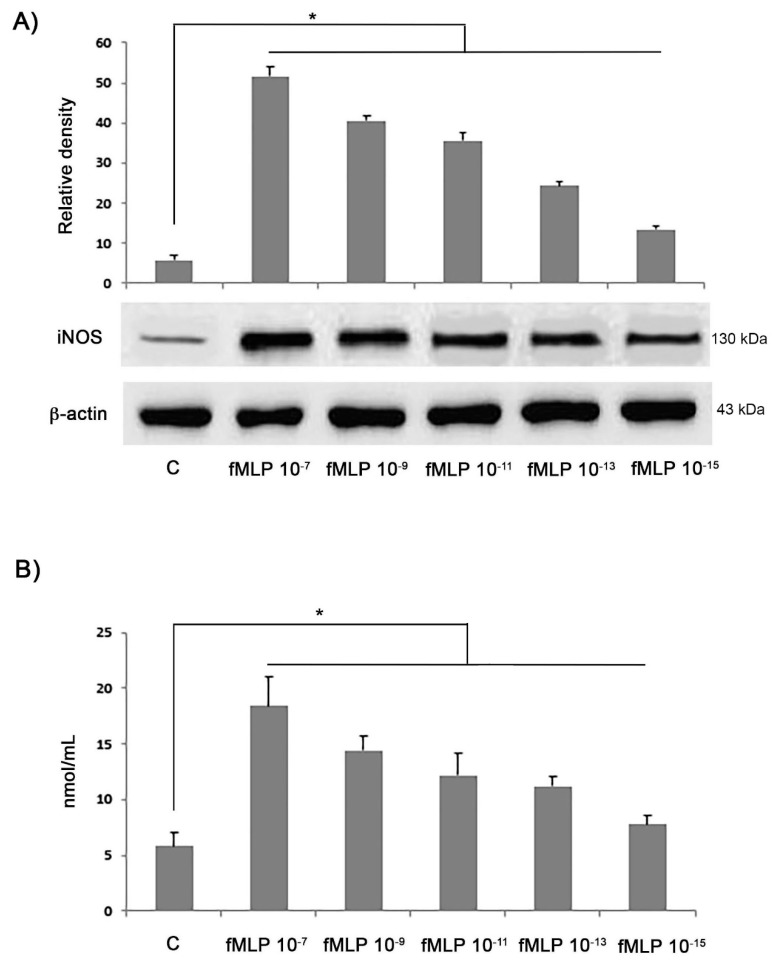
Immunoblotting detection of inducible nitric oxide synthase (iNOS) in controls and in neurons treated with different concentrations of *N*-formyl-l-methionyl-l-leucyl-phenylalanine (fMLP) for 48 h. β-actin was used as a loading control. (**A**) Densitometric analysis of iNOS expression is expressed as arbitrary units, after normalization against β-actin. (**B**) Nitric oxide production by fMLP-stimulated cell cultures performed with the Griess reaction, as described in the Experimental procedures section. Results are expressed as means ± SD of five experiments. * *p* < 0.05.

Stimulation of neuronal primary cultures with fMLP significantly increased the protein expression of inducible nitric oxide synthase (iNOS) in comparison to controls (*p* < 0.05), as well as the production of NO in the supernatant of cell cultures, in a dose-dependent manner, as reported in Figure 2A,B, respectively (*p* < 0.05 fMLP stimulated cells vs. controls). fMLP was concentration-dependently able to increase NO release from neuronal primary cultures, resulting in a 3-fold increase over the baseline at a concentration of 10^−7^ M (Figure 2B). Thus, the effects of fMLP on NO release were related to the increased iNOS expression, evaluated in neuronal primary cultures by immunoblotting reinforcing the concept that, under our in vitro experimental conditions, neurons respond to fMLP treatment with the same enzymatic activation machinery typical of pro-inflammatory stimuli.

### 3.3. Effects of the fMLP Treatment on Cell Apoptosis

**Figure 3:**
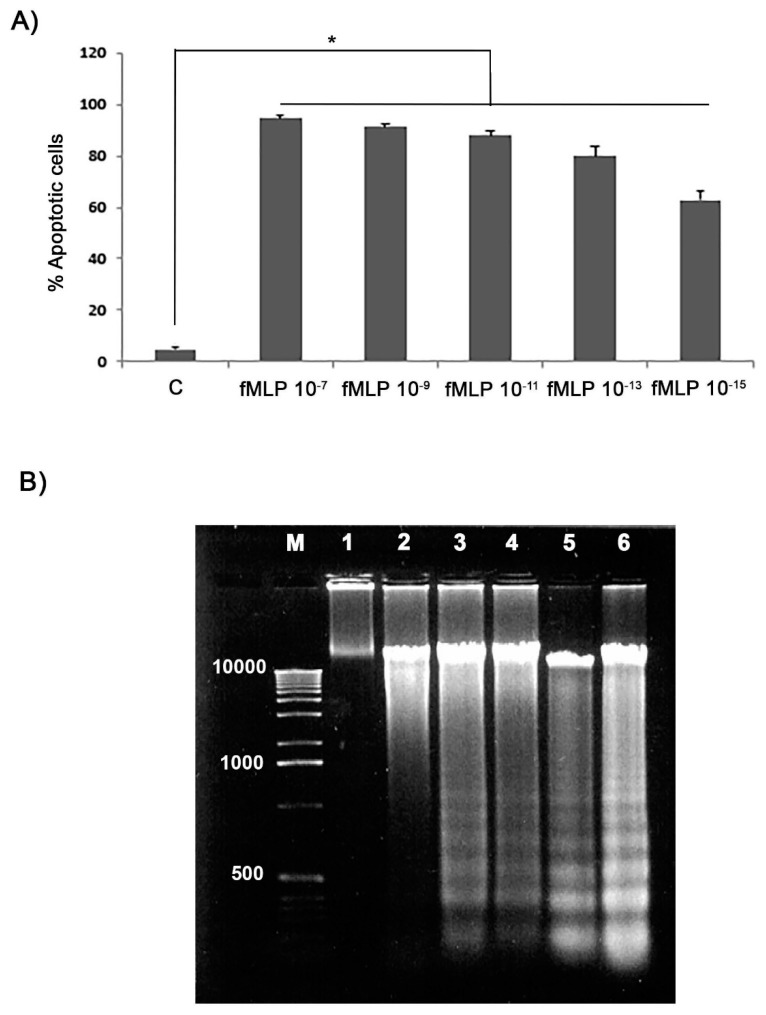
Effects of fMLP on the cell apoptosis percentage in cultured neurons. The percentage (%) of apoptotic cells was assessed using the Annexin V-CY3 assay apoptosis detection kit. (**A**) Cultured neurons were treated with different concentrations of fMLP for 48 h. Data are means ± SD of five experiments for each concentration. * *p* < 0.05. (**B**) DNA laddering demonstrating characteristic features of apoptosis after 48 h of fMLP treatment. 1 = control; 2 = fMLP 10^−7^ M 3 = fMLP 10^−9^ M; 4 = fMLP 10^−11^ M; 5 = fMLP 10^−13^ M; 6 = fMLP 10^−15^ M.

For the nuclear morphology analysis, cells stained with the Annexin V-CY3 apoptosis detection kit were observed in order to evaluate the pro-apoptotic effect of the fMLP treatment of neuron cultures. fMLP treatment of cell cultures was able to induce apoptosis in a dose-dependent manner. Cultures were treated with fMLP (10^−7^, 10^−9^, 10^−11^, 10^−13^, or 10^−15^ M) for 48 h. As shown in Figure 3A, fMLP (at 10^−7^ to 10^−15^ M) significantly induced apoptosis in comparison to control (*p* < 0.05). Results demonstrated that fMLP induced apoptosis in a dose-dependent manner: In fMLP-treated cells the percentage of apoptosis was almost 80% at 10^−13^ M (Figure 3A). A greater DNA fragmentation was also observed in fMLP-treated cells (for all concentrations tested) in comparison to controls (Figure 3B). In this regard, although the regulation of many important biological cell functions requires a relatively high concentration (>10^−7^ M) of fMLP [11,12], it is rare for nervous tissue to be exposed to such high concentrations due to its difficulty in crossing the blood–brain barrier. For this reason we decided to study the effect of fMLP at 10^−13^ M, which might be close to the physiologically relevant concentration of fMLP in CNS, as reported by other authors [13,14]. 

### 3.4. Molecular Markers of Apoptosis

**Figure 4:**
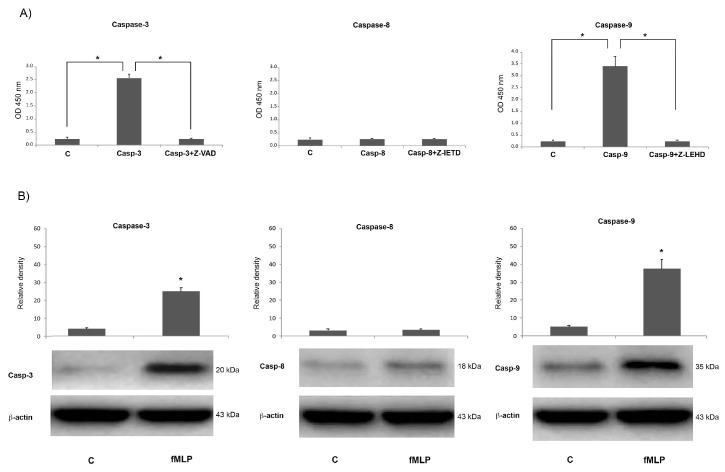
Caspase-3, -8 and -9 enzymatic activity in neurons. Untreated control cells (negative control). (**A**) Cells were treated with fMLP 10^−13^ M with or without pre-treatment with the caspase-3 inhibitor Z-valyl-alanyl-aspartic acid–fluoromethyl ketone (Z-VAD-FMK), caspase-8 inhibitor Z-Ile–Glu–Thr–Asp (IETD)-FMK or caspase-9 inhibitor Z-Leu–Glu–His–Asp (LEHD)-FMK. The y-axis shows the amount of free p-nitroanilide (pNA) released from the caspase-3, -8, and -9 specific substrates. Western blotting analysis of capases -3, -8 and -9 levels in fMLP-treated neurons. (**B**) Densitometric analysis of protein bands was expressed as arbitrary units, after normalization against β-actin. Data are representative of five experiments, and results are expressed as mean ± SD of five independent experiments. * *p* < 0.05 indicates significant differences.

A significantly higher caspase-3 enzymatic activity, determined by the CaspACE colorimetric assay, observed in fMLP-treated cells in comparison with untreated control cells (*p* < 0.05), was reported in Figure 4A (left). The activation was specific for caspase-3, as it was inhibited by the addition of the caspase-3 inhibitor Z-VAD-FMK. Moreover, to evaluate the apoptosis-initiating stages, the activity of both caspase-8 and caspase-9 was also evaluated. Figure 4A shows a significantly (*p* < 0.05) higher caspase-9 (right), but not caspase-8 (middle), enzymatic activity in fMLP-treated cells in comparison with untreated cells. The activation was specific for caspase-9, as it was inhibited by the addition of the specific inhibitor of caspase-9, namely Z-LEHD-FMK. The caspase activation was also evaluated by western blotting analysis with specific anti-caspase antibodies for the active subunits. Immunoblotting analysis showed that the expression of cleaved caspase-9, and cleaved caspase-3 was increased in fMLP-treated neurons, compared with the control group, whereas cleaved caspase-8 resulted expressed at comparable levels to those observed in control, as illustrated by densitometric analysis of visualized bands (Figure 4B). The results described above suggest that the increased expression of caspase-3 in fMLP-treated cells was attributable to the activation of caspase-9 but not of caspase-8.

### 3.5. fMLP Treatment Reduced the Level of Anti-Apoptosis Proteins and Increased the Level of Pro-Apoptosis Ones

**Figure 5:**
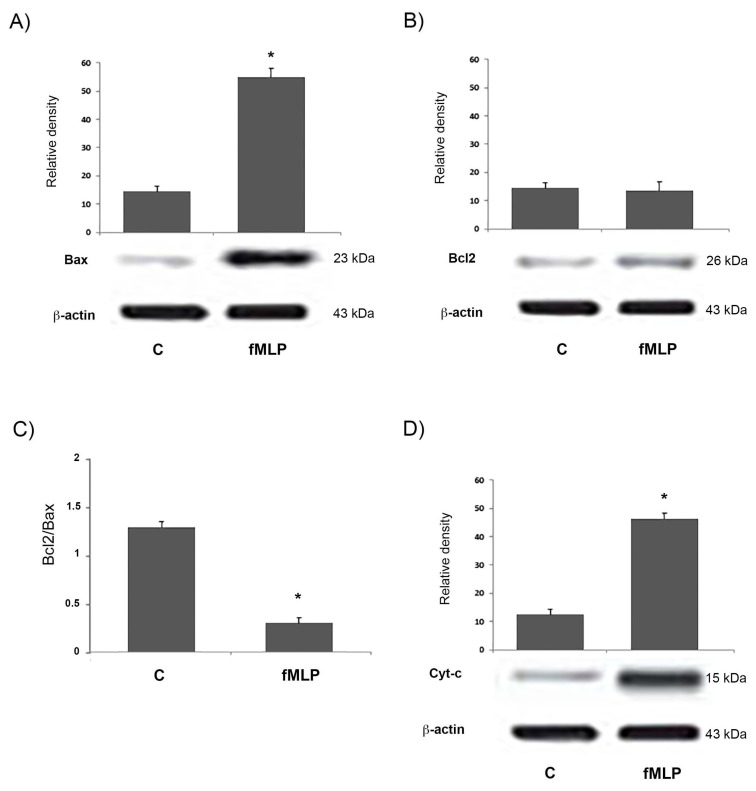
Western blotting analysis of Bax (**A**), Bcl-2 (**B**), and Cyt-c (**D**) in neurons treated with fMLP 10^−13^ M. The Bcl-2/Bax protein ratio (**C**) was evaluated by densitometric analysis of bands observed in the immunoblotting assay. Densitometric analysis of protein bands was expressed as arbitrary units, after normalization against β-actin (mean ± SD of five independent experiments). * *p* < 0.05 indicates significant differences.

We also evaluated the effect of the fMLP treatment on the apoptosis of neurons assaying the expression of apoptosis-related proteins. The Bcl-2 protein family drives the cell destiny, regulating the mitochondrial outer membrane integrity. This family comprises adaptor proteins containing the BH3 domain, such as Bim and Bid, able to initiate apoptosis, including prosurvival proteins, such as Bcl-2 and Bcl-xL. These last can inhibit the activation of a pro-apoptosis group consisting of Bax and Bak. The latter constitute the pore-like structures in the mitochondrial outer membrane, from which Cyt-c could be released and initiate the mitochondrial apoptosis pathway [15]. To evaluate a possible role for the anti-apoptotic protein Bcl-2 and the pro-apoptotic Bax in the apoptosis induced by fMLP, the expression of Bcl-2 and Bax was detected by western blotting. In this regard, as shown in Figure 5A,B, the expression of Bax increased with the fMLP treatment in comparison to untreated cells (*p* < 0.05), while the expression of anti-apoptotic protein Bcl-2 appeared similar to the control. Then, the Bcl-2/Bax ratio resulted significantly (*p* < 0.05) reduced in fMLP-treated cells compared to controls (Figure 5C). Furthermore, the cytosolic concentration of Cyt-c, which plays a key role in the caspase-dependent apoptotic pathway, also increased following fMLP treatment (Figure 5D).

These findings suggest that neuronal death observed in fMLP treatment might be associated to a mitochondria-dependent apoptosis pathway.

### 3.6. Effects of the iNOS Inhibition on the fMLP-Induced Cell Apoptosis

To investigate the role for NO in the apoptosis induced by fMLP treatment we evaluated the effect of the specific iNOS inhibitor 1400 W (25 μM, Alexis, Milan, Italy). In fMLP-stimulated cells.

**Figure 6:**
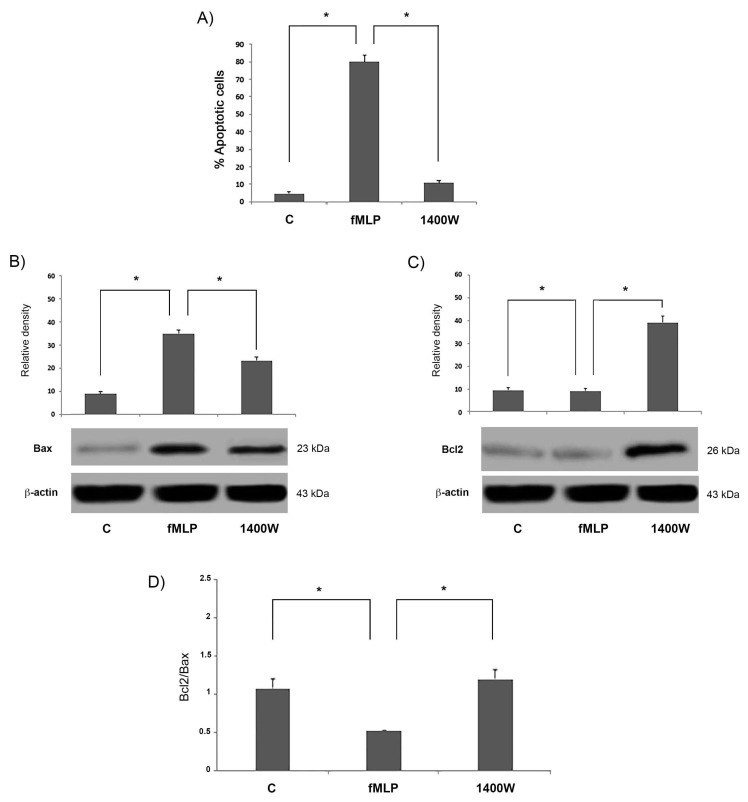
The percentage (%) of apoptotic cells was assessed using the Annexin V-CY3 assay apoptosis detection kit. (**A**) Cultured neurons were treated with fMLP 10^−13^ M alone or in presence of 1400 W for 48 h. Data are mean ± SD of five independent experiments for each concentration. * *p* < 0.05. (**B**,**C**) Immunoblotting detection of Bax and Bcl-2 in neurons treated with fMLP 10^−13^ M alone or in presence of the iNOS inhibitor, 1400 W, for 48 h. Densitometric analysis of protein bands was expressed as arbitrary units, after normalization against β-actin (mean ± SD of five independents experiments). The Bcl-2/Bax protein ratio expressed by densitometric analysis of bands observed in the immunoblotting assay. (**D**) Protein expression levels were normalized against β-actin. Values (means ± SD of five experiments) are expressed as arbitrary units. * *p* < 0.05 significantly different.

The percentage of apoptotic cells resulted in significantly reduced fMLP-stimulated cells in the presence of the NO inhibitor (*p* < 0.05) (Figure 6A). In this respect, we investigated the possibility that the iNOS inhibitor was able to interfere with Bcl-2 expression in treated cells. 

**Figure 7:**
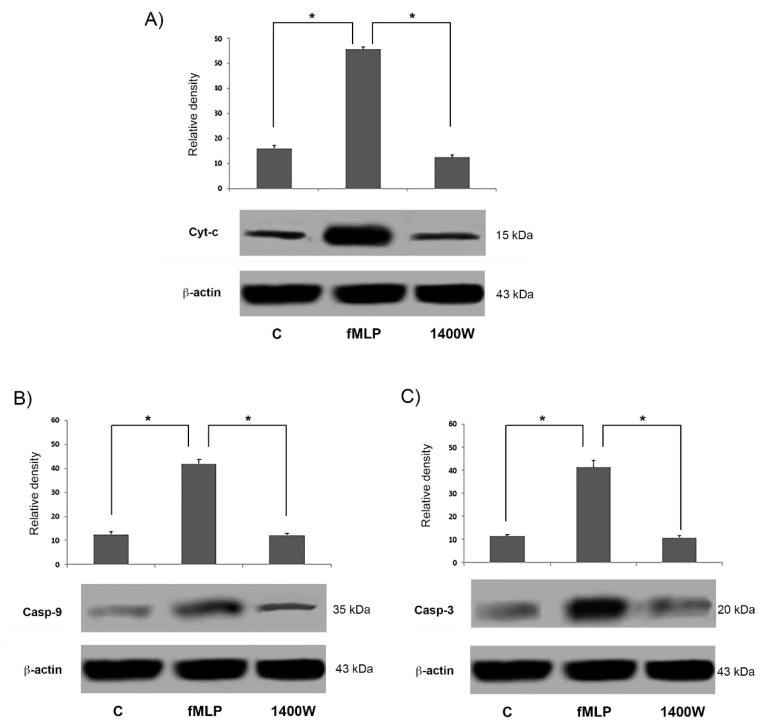
Western blotting analysis of apoptosis-related proteins in neurons treated with fMLP 10^−13^ M alone or in presence of 1400 W for 48 h. Densitometric analysis of protein bands expressed as arbitrary units, after normalization against β-actin (mean ± SD of five independent experiments). * *p* < 0.05 indicates significant differences. (**A**) Cyt-c, (**B**) caspase-9, and (**C**) caspase-3 levels.

As hypothesized, we observed in fMLP-stimulated cells that the presence of 1400 W led to a significant reduction (*p* < 0.05) of Bax expression, while conversely increasing Bcl-2 expression, thus significantly increasing the Bcl2/Bax ratio. This result suggests that NO release is involved in the regulation of apoptosis observed in fMLP-treated cells, possibly through the modulation of Bcl-2 expression (Figure 6B–D). Moreover, pre-treatment with 1400 W significantly (*p* < 0.05) attenuated the fMLP-induced increase of Cyt-c, cleaved caspase-9, and cleaved caspase-3 concentration, as reported in Figure 7. All these results demonstrate that the effects of apoptosis induction triggered by fMLP were probably mediated by NO generation.

## 4. Discussion

In this paper we demonstrated fMLP capacity to induce neuron apoptosis through NO-dependent caspase-9 activation. We found that fMLP treatment was able to increase the release of molecules (such as Cyt-c) from the mitochondrial compartment, which causes the activation of caspase-9 and downstream cleavage of caspase-3 [16]. 

Growing evidence shows that an important step in apoptosis is Cyt-c release from mitochondria. This is, in fact, an essential component of the complex that activates the death protease caspase-3 [17]. Moreover, we showed that fMLP-induced apoptosis is associated with Bcl-2 protein family modulation, since a significant increase of the Bcl2/Bax ratio in fMLP-treated neurons was detected. Interestingly, we also observed that iNOS inhibition reverted apoptosis, thus confirming a role for NO in regulation of the cell death program observed in fMLP-treated neurons. As demonstrated in this work, in fMLP-stimulated cells treatment with 1400 W reversed the up-regulation of Cyt-c, and cleaved caspase-9/3 in cells treated with fMLP, as well as the increased ratio of mitochondrion-mediated apoptosis regulating the Bcl2/Bax protein.

Overall, these results suggest that the release of NO from fMLP treated neurons induces apoptosis through the mitochondrial pathway and modulation of the Bcl-2 family proteins. Accumulated data indicate that physiologically relevant levels of NO play an important role in the apoptosis balance.

A cell may decide to undergo apoptosis process as a result of a shift balance between the anti-apoptotic and pro-apoptotic forces. In this delicate process the NO pathway is strictly linked to mitochondrial dysfunction [18]. 

In CNS, NO is involved in the regulation of different biological processes [19]. 

NO, a non-typical neurotransmitter, is important for maintenance of neural cell activities and normal brain functions. However, this molecule is able to induce positive or negative effects upon different signal pathways depending on cellular origins and locations. For example, oxidative injury to DNA, and activation of the DNA damage-sensing enzyme poly (ADP-ribose) polymerase (PARP) may induce neurotoxicity by production of NO through the iNOS activation. In this regard, mitochondria play a key role in the apoptotic cascades and cell death [20].

Inflammation is present in many diseases including those of the CNS, and is connected to mitochondrial dysfunction, as well as to the overproduction of oxidants, including nitric oxide [18]. 

Indeed, it was observed that in cerebral ischemia, traumatic brain injury, Parkinson’s disease, Huntington’s disease, Alzheimer’s disease and amyotrophic lateral sclerosis, NO is associated with inflammation, linked to mitochondrial dysfunction and thus conducive to neuronal death [21].

In this context, a growing number of observations has underlined that mitochondrial dysfunction, oxidative damage and chronic inflammation are common pathognomonic signs described in different neurodegenerative diseases [22].

Mitochondrial injury leads to the mitochondrial apoptosis pathway with the involvement of the Bcl-2 family members [23,24]. Due to the important role that mitochondria play in the apoptotic cascades and cell death [20], in our study we analysed the expression of mitochondrion-mediated apoptosis-associated proteins, evaluating the levels of Bax and Bcl-2, pro-apoptotic and anti-apoptotic proteins, respectively, since the Bax to Bcl-2 ratio plays an important role in the balance of cell apoptosis.

Our experimental data clearly showed that the expression of Bax protein was significantly increased in the fMLP-stimulated neurons.

In the mitochondrial apoptotic pathway, Bax acts downstream, since it leads to permeabilization of the mitochondrial outer membrane [25,26], considered an important key control able to switch the apoptotic process [27]. The permeabilization of the mitochondrial membrane leads to the release of Cyt-c, a component of the mitochondrial electron transfer chain, that after binding to Apaf-1 can induce caspase activation [26]. Then the Apaf-1-Cyt-c complex forms the apoptosome, which recruits procaspase-9 and initiates the formation of the caspase-9 holoenzyme, which in turn cleaves and activates downstream caspases, such as, caspase-3. In our experiments fMLP-treated neurons, show an increase of Cyt-c, indicating the activation of the mitochondrial apoptotic pathway. Growing evidence suggests that in neurodegenerative diseases, mitochondrial dysfunction and consequent apoptosis is an element that creates predisposition to the development of pathologies [18,28]. A particularly relevant aspect in neurodegenerative processes is the relationship between mitochondria and NO production. In CNS, the concentration of NO is a key factor for apoptosis balance. Predominantly, NO at low concentrations is neuroprotective and mediates physiological signaling, whereas higher concentrations of NO mediate neuroinflammatory actions resulting in neurotoxicity [29].

In the present work we found that N-formylated peptides are able to induce a robust production of NO in neuronal cells, which may lead to mitochondrial dysfunction culminating in the activation of a cell death program. 

In general, fMLP plays a key role in the immune response to infection and inflammation; it is considered a strong chemoattractant and activator of phagocytic cells in peripheral blood, leading to the release of oxygen-derived free radicals, which results in the removal of invading microorganisms [30].

It is well known that the in mouse FPR2 binds N-formyl peptides with low affinity and is constitutively expressed in different organs, such as the lung, liver, spleen and brain [8]. FPR2 activation has been proposed to play an anti-inflammatory role in neural signaling, although its functions has not yet been completely elucidated [8]. However, a number of studies have reported protective effects when FPR2 is engaged by its high affinity ligand, lipoxin, but no reports until now described the effects induced by the binding of FPR2 with formyl peptides. Interestingly, our results illustrate that fMLP may be selectively toxic to neurons by activating the intrinsic apoptosis pathway, thus indicating a link between infectious diseases and neurodegeneration. 

Since fMLP may easily reach CNS through circumventricular organs [4] during chronic peripheral infectious diseases when there is persistent circulating fMLP, it could be a potential risk for neurons. 

The present study emphasizing the potential role of infectious agents, such as N-formyl peptides, in neurodegenerative diseases may help to promote the development of new therapies able to modulate the expression of the N-formyl peptide receptors.
